# Modulation of Gut Microbial Community and Metabolism by Dietary Glycyl-Glutamine Supplementation May Favor Weaning Transition in Piglets

**DOI:** 10.3389/fmicb.2019.03125

**Published:** 2020-01-28

**Authors:** Yiqin Yan, Baoyang Xu, Boqi Yin, Xiaofan Xu, Yaorong Niu, Yimei Tang, Xinkai Wang, Chunlin Xie, Tao Yang, Shuyi Zhou, Xianghua Yan, Libao Ma

**Affiliations:** ^1^College of Animal Science and Technology, Huazhong Agricultural University, Wuhan, China; ^2^The Cooperative Innovation Center for Sustainable Pig Production, Huazhong Agricultural University, Wuhan, China; ^3^Hubei Provincial Engineering Laboratory for Pig Precision Feeding and Feed Safety Technology, Wuhan, China

**Keywords:** Gly-Gln, gut microbiota, microbial metabolites, 16S rDNA, weaning transition

## Abstract

Gut microbiota plays a crucial role in diet nutrient metabolism and maintaining host health. The synthetic dipeptides glycyl-glutamine (Gly-Gln) used as diet supplementation to improve the weaning transition of newborns could be metabolized by certain bacteria *in vitro*. However, the effect of diet Gly-Gln supplementation on gut microbiota *in vivo* remains largely unknown. 240 piglets at the age of 28 days (day 28) were randomly assigned to two groups that received a basal diet (Ctrl group) or a basal diet supplemented with 0.25% Gly-Gln (Gly-Gln group) for 3 weeks. Five piglets from each group were euthanized for sampling after overnight fasting on day 38 and day 49, respectively. We determined their structure shifts of the gut microbiota using 16S rDNA-based high-throughput sequencing analysis. Microbial metabolites short-chain fatty acids (SCFAs) in the ileum and the colon were determined with high-performance gas chromatography. The concentrations of endocrine peptides including epidermal growth factor, glucagon-like peptide-1, and glucagon-like peptide-2 in ileal mucosa, as well as the serum concentration of interleukin 1 beta, interleukin 6, interleukin 10, and tumor necrosis factor alpha were determined using Enzyme-Linked Immunosorbent Assay. In addition, we also checked the diarrhea ratio, growth performance, and intestinal morphology to assess the favorable effect of dietary Gly-Gln supplementation during the weaning transition. Dietary Gly-Gln supplementation beneficially altered the gut microbiota by increasing bacterial loading, elevating alpha diversity, and increasing the relative abundance of anaerobes and fiber-degrading bacteria (Phylum Fibrobacteres). Accordingly, the microbial metabolites SCFAs in both colon and ileum, as well as the downstream endocrine peptides in the ileum increased. Meanwhile, dietary Gly-Gln’s favorable weaning transition was reflected in the increase of growth performance indices and the reduced inflammatory response in a time dependent manner. There were significant correlations among the bacteria which responded to dietary Gly-Gln supplementation and these checked indices. Taken together, dietary Gly-Gln supplementation selectively modulated the gut microbiota, which may favor piglets’ weaning-transition. These findings suggest that gut microbiota targeted approaches can be potentially used to improve weaning transition of piglets by dietary functional amino acid.

## Introduction

Trillions of diverse microbes reside in intestinal lumen and constitute a complex and mostly anaerobic ecosystem namely gut microbiota that play a crucial role in maintaining host health (Martin et al., 2010; Chassard et al., 2012). Gut microbiota is generally recognized as indispensable in preventing pathogen infection, maintaining intestinal function, and regulating the immune response and inflammation (Oliphant and Allen-Vercoe, 2019). In addition, gut microbiota engages in the catabolism of the three dietary macronutrients (carbohydrates, proteins, and fat) that reach the colon upon either escaping primary digestion or resisting primary digestion (Oliphant and Allen-Vercoe, 2019). The microbial metabolites of those substrates do not only serve as nutrients useable by the host, but also act as mediators to modulate the physiology and gene expression of host cells (Micah et al., 2007; Belkaid and Hand, 2014). On the other hand, the dysbiosis of gut microbiota in turn leads to the host’s dysfunction, even disease (Kim et al., 2014; Gillis et al., 2018; Soderborg et al., 2018). Weaning as a critical event in the pig’s lifecycle is frequently accompanied with severe gut microbiota dysbiosis induced by abrupt changes in the diet and environment (Gresse et al., 2017). Numerous studies conducted about weaning transition have reported a decrease in bacteria of the *Lactobacillus* group and a loss of microbial diversity, whereas *Clostridium* spp., *Prevotella* spp. or facultative anaerobes such as *Proteobacteriaceae*, including *Escherichia coli* increased (Gresse et al., 2017). Increasing evidence reveals that dietary organic acids, essential oils, and prebiotics can benefit the gut microbiota of piglets in weaning transition by increasing helpful bacteria and reducing harmful ones (Gong et al., 2008; Jiao et al., 2014; Zeng et al., 2015). Thus, improving gut microbiota has been suggested as a potential approach to favoring piglets’ weaning transition (Gresse et al., 2017).

The three main dietary macronutrients carbohydrates, proteins, and fats can significantly affect the composition of gut microbiota (David et al., 2013; Oliphant and Allen-Vercoe, 2019). In addition to the well-studied carbohydrates and fats, dietary supplementation with peptides and amino acids have been increasingly proved to shift gut microbiota (Yang et al., 2016; Oliphant and Allen-Vercoe, 2019). It has been reported that dietary supplementation with functional amino acids can shift the composition of the intestinal microbiota of animals, such as arginine and leucine in mice (Wada et al., 2013; Ren et al., 2014), cysteine in piglets (Xu et al., 2014), cysteine in piglets (Xu et al., 2014), L-glutamine (Gln) in rabbits (Chamorro et al., 2010). To our knowledge, there was no direct evidence for the effect of dietary supplementation with glycyl-glutamine (Gly-Gln) on the gut microbiota of post-weaned piglets although it has been used to improve weaning stress (Jiang et al., 2000, 2009).

Gly-Gln can decompose to release Gln *in vivo*, which improves intestinal barrier (Jiang et al., 2011). Gln is a well-studied non-essential amino acid (NEAA), and a major metabolic resource for the intestinal epithelial cell (Lallès et al., 2007). Besides Gln’s generally known key role in the maintenance of intestinal structure and function, it can also be metabolized by the anaerobic organism micrococcus aerogenes to volatile acids including acetic acid, butyric acid, and carbon dioxide *in vitro* (Horler et al., 1966). The rapidity of Gln fermentation has been proved evident in fecal incubations trial when the amino acid was incubated as sole N-source *in vitro* (Smith and Macfarlane, 1997). Chamorro et al. demonstrated that *in vivo*, 1.0% Gln supplementation to the diets of post-weaned rabbits decreased fattening mortality and modified intestinal microbiota (Chamorro et al., 2010). Furthermore, Gln plays an important role in nitrogen balance and protein synthesis in resident bacteria of the small intestine (Dai et al., 2013). In addition, Zambom de Souza et al.’s (2015) pilot study showed that oral supplementation with Gln alters the gut microbiota of obese and overweight adults. These results indicate that Gln can regulate gut microbiota, which may contribute to its beneficial effect on the host (Fan et al., 2015). Nevertheless, the effect of dietary Gly-Gln supplementation on the gut microbiota of piglets during weaning transition remains unknown. Here, we determined the effects of dietary Gly-Gln supplementation on the gut microbiota of post-weaned piglets.

## Materials and Methods

### Experimental Design and Animals

Our animal experiment was carried out in accordance with the Protocol of Hubei Province Laboratory Animal Management and with the approval of the Institutional Animal Care and Use Committee (IACUC) of Huazhong Agricultural University (Wuhan, China). All efforts were made to minimize animal suffering. 240 piglets crossbred healthy piglets of 28 days old [(Duroc × Landrace × Yorkshire), female and male in half, initial body weight 7.6 ± 0.04 kg] were evenly and randomly assigned to control group (Ctrl) and glycyl-glutamine group (Gly-Gln) with similar body weight. Ctrl group was fed the basal diet while Gly-Gln group fed the basal diet supplemented with 0.25% glycyl-glutamine (reagent grade, ≥ 99%, Hubei Huntide Biotech Co., Ltd). The basal diet was formulated to meet the requirements of the pigs (National Research Council [NRC], 2012, Supplementary Table S1), and glycine was used to formulate isonitrogenous diets as previously described (Wang et al., 2008). The amounts of supplemental Gly-Gln used in this study were based on a previous study (Li et al., 2003; Jiang et al., 2009). Diets and water were supplied *ad libitum*.

The experiment lasted 21 days (from day 28 to day 49). On day 38 and day 49, five piglets from each group were selected randomly and then euthanized for sampling after fasting overnight. Blood samples (10 ml) were collected and then centrifugated for 10 min (3,500 × *g*, 4°C) to obtain serum samples (Zhou et al., 2017). Immediately after slaughter, intestinal contents from ileum, colon and rectum were collected and placed in liquid nitrogen, and the whole small intestine was rapidly removed and then washed with ice-cold PBS. Three 2-cm long segments of jejunum were removed from the middle portion of the small intestine and rinsed with ice-cold PBS, then fixed in 4% paraformaldehyde solution for measuring intestinal morphology (Yang et al., 2014).

We also checked the growth performance of piglets. The piglets were weighed at the beginning (day 28) and the end (day 49) to determine their average daily gain (ADG). At the same time, we recorded the average daily feed intake (ADFI). To determine diarrhea ratio, the fecal score was checked daily using a scale: 1 = solid and cloddy, 2 = soft with shape, 3 = very soft or viscous liquid, and 4 = watery or with blood (Barba-Vidal et al., 2017). Then, diarrhea rate (DR) was determined according to the above description with a threshold of the fecal score value 3 (Chen et al., 2013).

### Gut Microbiota Profiling

The total genomic DNA of fecal bacteria (stool from the rectum) was extracted using the protocol of the Repeated Bead Beating Plus Column (RBB + C) Method (Costea et al., 2017). The integrity of DNA was assessed by agarose gel electrophoresis. The genomic DNA was used as a template for PCR amplification. Universal primers 338F and 806R were used for PCR amplification of the V3–V4 hypervariable regions of 16S rRNA genes (338F,5′-ACTCCTACGGGAGGCAGCA-3′; 806R, 5′-GGACTACHVGGGTWTCTAAT-3′) (Fadrosh et al., 2014). Sequencing was performed with an Illumina MiSeq PE300.

To obtain more accurate and reliable results in subsequent bioinformatics analysis, raw data were cleansed by the in-house procedure (Fadrosh et al., 2014). Then paired end reads with overlap were merged to tags. The high-quality paired-end reads were combined to tags based on overlaps with FLASH (Magoč and Salzberg, 2011). The tags were then clustered to OTU (Operational Taxonomic Unit) by scripts of software USEARCH (v7.0.1090) (Edgar, 2013). After that, they were clustered into OTU with a 97% threshold using UPARSE, and the OTU unique representative sequences were obtained; Chimeras were filtered out by UCHIME (v4.2.40); OTU representative sequences were taxonomically classified using Ribosomal Database Project (RDP) Classifier v.2.2 trained on the database Greengene_2013_5_99 (DeSantis et al., 2006) with 0.6 confidence values as cutoff. The alpha diversity indices including observed species value, chao1 value, ACE value, and Shannon value are calculated by Mothur (v1.31.2) (Schloss et al., 2009) with the corresponding rarefaction curve was drawn by software R (v3.5.1). Phylogenetic beta diversity measures such as unweighted UniFrac distance metrics analysis and principal-component analysis (PCoA) were done using the Quantitative Insights into Microbial Ecology 2(QIIME 2) (Bolyen et al., 2019).

### Gut Microbial Metabolites

We determined the short-chain fatty acids (SCFAs) including acetic acid, propionic acid, butyric acid, valeric acid, isobutyric acid, and isovaleric acid in the digesta of ileum and colon using gas chromatography (GC) with a modification of the previous method (Franklin et al., 2002). In brief, 1 *g* of the digesta samples was weighed into a 2 ml centrifuge tube with 1 ml of methanol added. After being vortexed for 30 s, the sample was centrifuged for 10 min (12,000 *g*, 4°C). The supernatant (1 ml) was transferred into centrifuge tubes (2 ml) and mixed with 0.2 ml 25% metaphosphoric acid. After 30 min at 4°C, the tubes were centrifuged for 10 min (12,000 *g*, 4°C) again. Aliquots of the supernatant (1 ml) were analyzed using GC method.

### Endocrine Peptides Levels in the Ileal Mucosa

The determination of glucagon-like peptide-1 (GLP-1), glucagon-like peptide-2 (GLP-2), and epidermal growth factor (EGF) levels in intestinal mucosa was conducted using Enzyme-Linked Immunosorbent Assay (ELISA) kits as the instructions described (Cusabio Biotech Co., Hubei, China). In brief, 100 mg tissue was rinsed with 1 × PBS, homogenized in 1 ml of 1 × PBS and stored overnight at −20°C. After two freeze-thaw cycles were performed to break the cell membranes, the homogenates were centrifuged for 5 min (5,000 × *g*, 4°C). The supernatant was removed and assayed immediately.

### Serum Cytokines Indices Levels

Serum samples were thawed and thoroughly mixed immediately before analysis. The levels of serum cytokines including interleukin 1 beta (IL-1β), interleukin 6 (IL-6), interleukin 6 (IL-10), and tumor necrosis factor alpha (TNF-α) were measured using ELISA kits (Cusabio Biotech Co., Hubei, China). In brief, 100 μl serum was thawed at 4°C and then was centrifuged for 5 min (5,000 × *g*, 4°C). The supernatant was removed and assayed immediately.

### Histopathological Examination of Jejunum

The sections of jejunum were dehydrated, paraffin sectioned, and hematoxylin-eosin stained, then observed under the microscope to measure villus height and crypt depth. Histological slices were examined with an Olympus BX51 microscope with Integrated Digital Imaging Analysis System (Olympus Corporation, Tokyo, Japan). Morphometric parameters of intestinal architecture were observed using 4× images. Villus height and crypt depths were measured manually. The villus height was defined by the vertical distance from the crypt opening to the tip of the villus. The crypt depth was defined from the base of the crypt to the level of the crypt opening (Li et al., 1990).

### Growth Performance of Piglets

We monitored the growth performance of piglets by determining the values of ADG, ADFI, and DR. The body weights of piglets on day 28, day 38, and day 49 were determined in the morning before being fed. At the same time, the amount of the feed consumed by each group during the experiment was recorded. ADG, FCR, and DR were calculated as follows: ADG = Body weight gain/(Number of Piglets × days); ADFI = Diet intake/(Piglets number × Days); DR = Number of diarrhea piglets/(Piglets number × Days).

### Statistical Analysis

Results were presented as mean ± SEM. Experimental data were analyzed by one-way ANOVA and the Duncan multiple comparison test with GraphPad 8.0 software. Significance was presented as ^∗^*P* < 0.05, and ^∗∗^*P* < 0.01, whereas *P* values between 0.05 and 0.10 were considered as indicative of a trend.

## Results

### Effects of Dietary Gly-Gln Supplementation on the Distribution of OTUs

**FIGURE 1:**
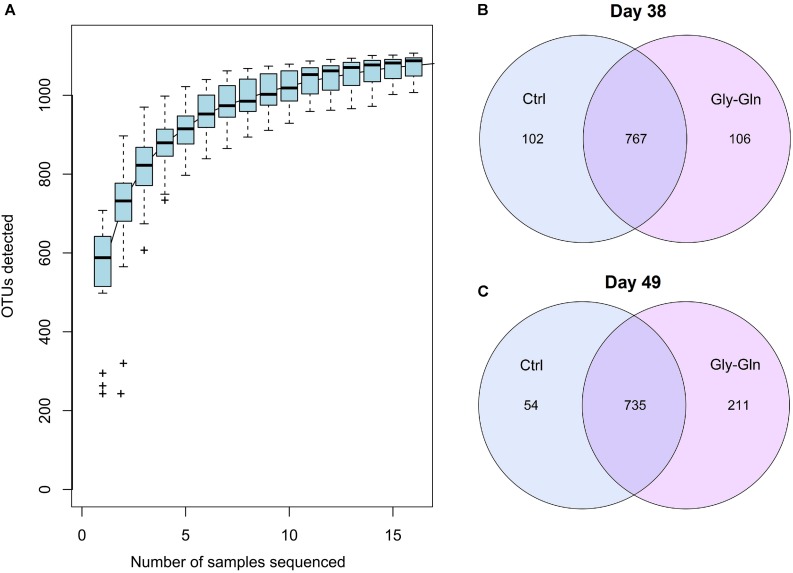
The common/unique OTUs number between Gly-Gln group and Ctrl group. Species accumulation curves based on OTU **(A)**. Common/unique OTU number distribution on day 38 **(B)** and day 49 **(C)** (*n* = 5).

We conducted 16S rDNA amplicon high-throughput sequencing to profile gut microbiota composition. On day 38, 67,094 ± 357 reads were harvested from the control and 67,196 ± 238 reads were harvested from the Gly-Gln group. On day 49, 67,192 ± 665 reads were harvested from the control and 66,756 ± 635 reads were harvested from the Gly-Gln group. OTUs were obtained at a sequence-similarity level of 97%. To accesses the sampling bias, we plotted species accumulation curves obtained using all the OTU data and confirmed that more than 90% of the OTUs were sampled at the asymptote (Figure 1A). We then used a Venn diagram to show shared and unique OTUs number. On day 38, there were 767 common OTUs, 102 unique OTUs in the control group and 106 unique OTUs in the Gly-Gln group (Figure 1B). On day 49, the number of common OTUs decreased to 735, while the unique OTUs in the control group decreased to 54 and markedly increased to 211 in the Gly-Gln group (Figure 1C).

### Effects of Dietary Gly-Gln Supplementation on Diversity of the Gut Microbiota

**FIGURE 2:**
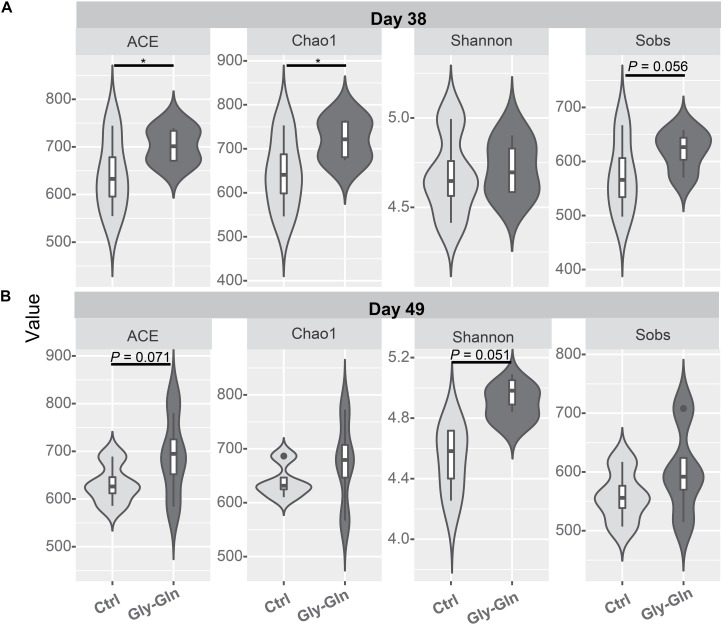
Effects of dietary Gly-Gln supplementation on alpha diversity of the gut microbiota in piglets. The alpha diversity indices ACE, Chao1, Shannon, and Sobs on day 38 **(A)** and day 49 **(B)** respectively. Significance was presented as ^∗^*P* < 0.05, whereas *P* values between 0.05 and 0.10 were considered as indicative of a trend (*n* = 5).

**FIGURE 3:**
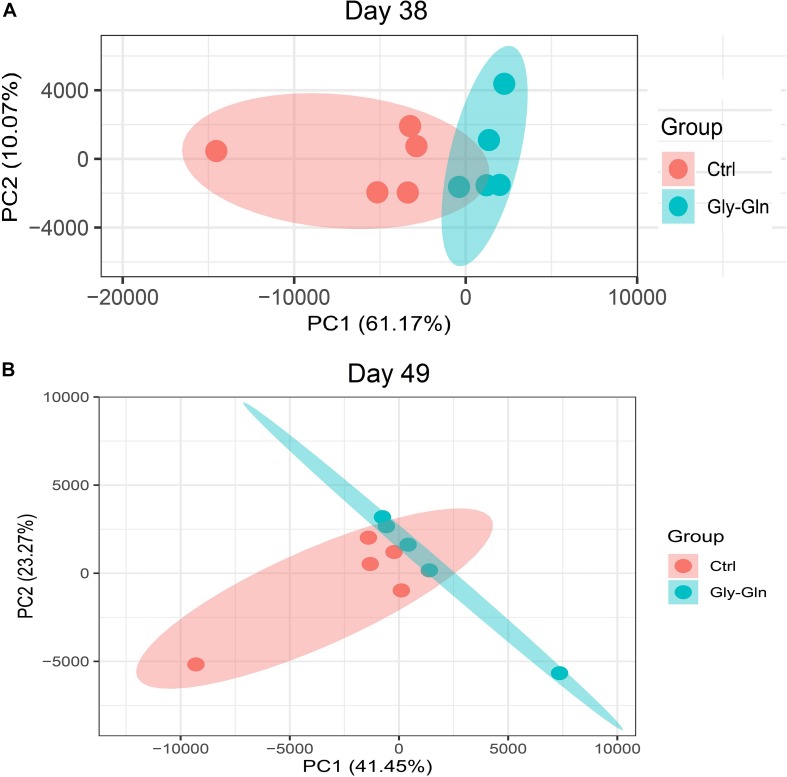
PCoA of gut microbiota between the Ctrl and Gly-Gln group (*n* = 5). **(A)** PCoA of gut microbiota on day 38; **(B)** PCoA of gut microbiota on day 49. The PC1 and PC2 in the axis indicated the top 2 percentage of gut microbiota variation explained by dietary Gly-Gln supplementation treatment.

Dietary Gly-Gln supplementation altered the diversity of the piglets’ gut microbiota. Alpha diversity indices indicate the diversity of gut microbiota in a single sample. Figure 2A showed that on day 38, supplementation with Gly-Gln increased the alpha diversity indices ACE (*P* < 0.05) and Chao 1 (*P* < 0.05); Sobs in Gly-Gln group tended to be higher than that of the Ctrl group (*P* = 0.056). On day 49, Gly-Gln had a tendency to increase the ACE (*P* = 0.071) and Shannon (*P* = 0.051) (Figure 2B). We then determined the Beta diversity between Gly-Gln group and Ctrl group with Principle coordinate analysis (PCoA). As shown in Figure 3, the PCoA plot revealed that the gut microbiota was markedly altered on day 38 (Figure 3A) as well as on day 49 (Figure 3B), making it evident that Gly-Gln group formed a distinct cluster markedly away from that of the Ctrl group.

### Effects of Dietary Gly-Gln Supplementation on Abundance of the Gut Microbiota

**FIGURE 4:**
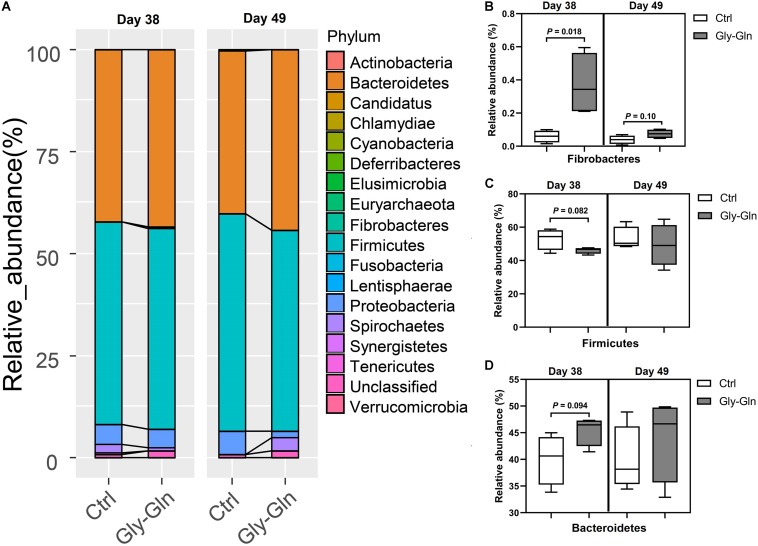
Effect of dietary Gly-Gln supplementation on the relative abundance of gut microbiota at phylum level. **(A)** The relative abundance of gut microbiota at phylum-level on day 38 and day 49 respectively. The relative abundance change of Fibrobacteres **(B)**, Firmicutes **(C)**, and Bacteroidetes **(D)** in gut microbiota. The values are expressed as means ± SEM, *n* = 5.

The changes in bacterial composition were determined at different taxonomic levels. As shown in Figure 4A, we observed a significantly shift at phylum level. In detail, the relative abundance of Fibrobacteres in Gly-Gln group increased than in the Ctrl group on day 38 (0.37% VS. 0.059%, *P* = 0.018); this increase was reduced on day 49 (0.075% VS. 0.039%, *P* = 0.10) (Figure 4B). Figure 4C showed that the relative abundance of Firmicutes have a tendency to be lower in Gly-Gln group than in the Ctrl group on day 38(46.09% VS. 52.97%, *P* = 0.082). On the contrary, the relative abundance of Bacteroidetes have a tendency to be higher in the Gly-Gln group than in the Ctrl group (45.42% VS. 40.02%, *P* = 0.094) (Figure 4D). The differences in the relative abundances of neither Firmicutes nor Bacteroidetes were significant on day 49 (Figures 4C,D).

**FIGURE 5:**
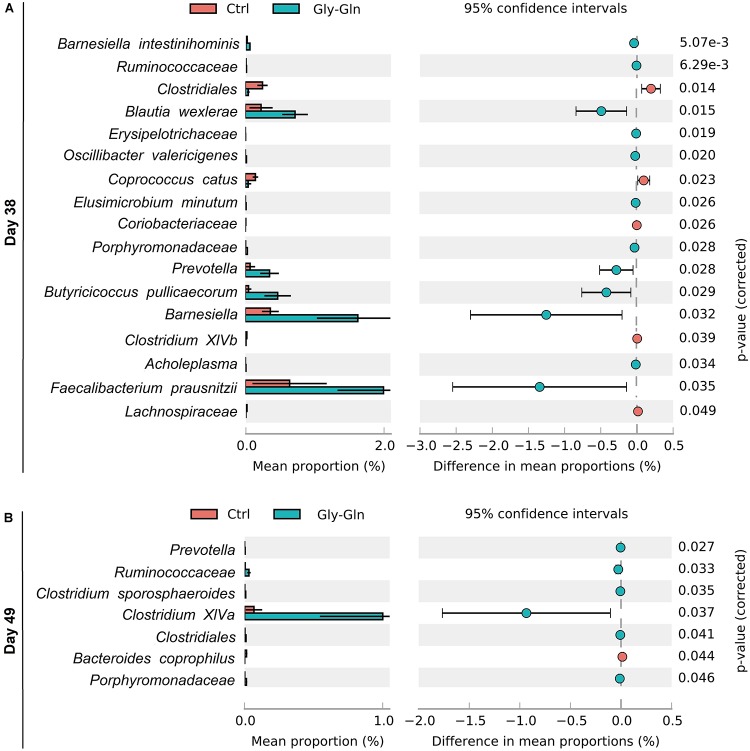
Effect of dietary Gly-Gln supplementation on the relative abundance of gut microbiota at species-level. The relative abundance of different species on day 38 **(A)** and day 49 **(B)** respectively.

To gain insights into species which responded to dietary Gly-Gln supplementation, Welch’s two-sided *t*-test at species-level was performed with software STAMP (Parks et al., 2014). We identified 17 species on day 38 and 7 species on day 49, respectively (Figures 5A,B). On day 38, there were 12 species with higher relative abundance in Gly-Gln group than in Ctrl group including *Barnesiella intestinihominis*, *Ruminococcaceae*, *Erysipelotrichaceae*, *Oscillibacter valericigenes*, *Elusimicrobium minutum*, *Porphyromonadaceae*, *Prevotella*, *Butyricicoccus pullicaecorum*, *Barnesiella*, *Acholeplasma*, and *Prausnitzii*. On the contrary, there were 6 species with lower relative abundance in Gly-Gln group than in Ctrl group including *Clostridiales*, *Blautia wexlerae*, *Coprococcus catus*, *Coriobacteriaceae*, *Clostridium XlVb*, and *Lachnospiraceae*. Then on day 49, there were 6 species (*Prevotella*, *Ruminococcaceae*, *Clostridium XlVa*, *Clostridiales*, and *Porphyromonadaceae*) with a higher relative abundance and only one species *Bacteroides coprophilus* with lower relative abundance in Gly-Gln group than in Ctrl group.

### Effects of Dietary Gly-Gln Supplementation on SCFAs Concentration in the Digesta of Ileum and Colon

To explore the metabolic alterations associated with dietary Gly-Gln supplementation, the metabolites in the digesta from ileum and colon were determined. As shown in Table 1, compared to Ctrl group, dietary Gly-Gln supplementation increased the concentration of propionic acid (*P* = 0.005), Butyric acid (*P* = 0.036), and valeric acid (*P* = 0.007) on day 38, and butyric acid (*P* = 0.008) on day 49 as well in colonic digesta of piglets from Gly-Gln group. In ileum digesta, dietary Gly-Gln supplementation increased the concentration of propionic acid (*P* = 0.045), valeric acid (*P* = 0.013), and isovaleric acid (*P* = 0.069) on day 38, and also butyric acid (*P* = 0.002) and isobutyric acid (*P* = 0.051) on day 49.

**TABLE 1:** The concentration of SCFAs in the digesta of colon and ileum.

**Item(μ mol/g)**	**Colon**			**Ileum**		
	**Ctrl**	**Gly-Gln**	***P*-value**	**Ctrl**	**Gly-Gln**	***P*-value**
**Day 38**						
Acetic acid	7.19 ± 1.50	6.96 ± 0.74	0.804	5.6 ± 0.56	5.11 ± 0.69	0.207
Propionic acid	3.67 ± 0.54	5.43 ± 0.68	0.005	1.75 ± 0.29	2.57 ± 0.72	0.045
Butyric acid	0.34 ± 0.09	0.47 ± 0.03	0.036	0.18 ± 0.06	0.23 ± 0.09	0.336
Isobutyric acid	3.54 ± 0.67	4.20 ± 0.33	0.138	2.36 ± 0.21	2.55 ± 0.18	0.149
Valeric acid	0.45 ± 0.04	0.58 ± 0.05	0.007	0.35 ± 0.035	0.42 ± 0.03	0.013
Isovaleric acid	0.48 ± 0.05	0.56 ± 0.25	0.556	0.32 ± 0.06	0.38 ± 0.02	0.069
**Day 49**						
Acetic acid	6.54 ± 1.60	6.17 ± 1.25	0.791	4.22 ± 0.33	4.55 ± 0.85	0.447
Propionic acid	2.13 ± 0.36	2.49 ± 0.26	0.120	1.39 ± 0.28	1.52 ± 0.26	0.444
Butyric acid	0.95 ± 0.21	1.49 ± 0.28	0.008	0.59 ± 0.07	0.82 ± 0.09	0.002
Isobutyric acid	0.18 ± 0.04	0.21 ± 0.03	0.140	0.09 ± 0.02	0.11 ± 0.00	0.051
Valeric acid	0.27 ± 0.06	0.34 ± 0.05	0.074	0.17 ± 0.03	0.20 ± 0.03	0.105
Isovaleric acid	0.20 ± 0.06	0.25 ± 0.05	0.123	0.11 ± 0.09	0.15 ± 0.03	0.404

### Effects of Dietary Gly-Gln Supplementation on the Concentration of Endocrine Peptides in the Ileal Mucosa

Table 2 showed the effects of dietary Gly-Gln supplementation on the concentration of endocrine peptides in the ileal mucosa. On day 38, compared with Ctrl group, the concentration of GLP-1 (*P* = 0.002), GLP-2 (*P* = 0.004), and EGF (*P* = 0.044) were significantly higher in Gly-Gln group. On day 49, compared with Ctrl group, the concentration of GLP-2 (*P* = 0.037) in Gly-Gln was also significantly higher, and the concentration of EGF (*P* = 0.055) tended to be higher.

**TABLE 2:** The concentrations of endocrine peptides in ileal mucosa of piglets on the day 38 and day 49.

**Item**	**Ctrl**	**Gly-Gln**	***P*-value**
**Day 38**			
GLP-1 (pg/g)	1.770.06	2.070.14	0.002
GLP-2(pg/g)	3.560.42	4.700.49	0.004
EGF (ng/g)	187.5516.86	213.5317.69	0.044
**Day 49**			
GLP-1 (pg/g)	2.490.48	2.940.35	0.128
GLP-2(pg/g)	3.670.87	5.851.76	0.037
EGF (ng/g)	200.9513.47	224.0418.59	0.055

### Effects of Dietary Gly-Gln Supplementation on the Concentration of Cytokines in the Serum

As shown in Table 3, compared with control group, dietary Gly-Gln supplementation reduced serum IL-1β(*P* = 0.032) and TNF-α (*P* = 0.037) on day 38, as well as IL-1β (*P* = 0.002) on day 49 (*P* = 0.002). There was increase in the concentration of IL-6(*P* = 0.004) and IL-10(*P* = 0.003) in Gly-Gln group on day 49, but not on day 38.

**TABLE 3:** The concentration of serum cytokines in the piglets.

**Item (pg/ml)**	**Ctrl**	**Gly-Gln**	***P*-value**
**Day 38**			
IL-1β	48.914.39	39.136.18	0.032
TNF-α	369.6521.61	334.1718.31	0.037
IL-6	276.3431.16	277.4828.98	0.959
IL-10	153.357.47	144.399.98	0.188
**Day 49**			
IL-1β	40.271.86	31.513.39	0.002
TNF-α	309.3828.48	274.2319.56	0.076
IL-6	252.1338.24	173.3110.24	0.004
IL-10	127.564.50	156.8413.00	0.003

### Effects of Dietary Gly-Gln Supplementation on the Jejunal Epithelial Integrity and Growth Performance of Piglets

We re-evaluated the beneficial effect of dietary Gly-Gln supplementation on the weaning transition of piglets. Supplementary Figure S1 showed the increased gap between the intestinal glands and hyperplastic glands observed in control group was ameliorated in groups supplemented with Gly-Gln on day 38. Gly-Gln group showed an improved jejunum epithelium morphology than Ctrl group on day 49. As shown in Supplementary Table S2, dietary Gly-Gln supplementation more notably elevated the villus height compared with the control on day 38 (*P* = 0.029) and day 49 (*P* = 0.0036). This improved result was also observed for crypt depth on day 38 (*P* = 0.0024). The increase of villus height/crypt depth ratio lasted from day 38 (*P* = 0.0069) to day 49 (*P* = 0.0002).

The growth performance was shown in Supplementary Table S3, Evidently, supplementation with Gly-Gln more significantly increased terminal BW compared to the control after 21 days (*P* = 0.018). The tendency of this BW promotion effect was already observed on day 38 (*P* = 0.072). Piglets’ ADFI in Gly-Gln group was significantly higher than in Ctrl group (*P* < 0.05). Similarly, the ADG of this group was significantly higher than Ctrl groups (*P* < 0.05). Dietary supplementation with Gly-Gln trended to reduce the diarrhea incidence from day 28 to day 49 (*P* = 0.053) more effectively compared to the control.

### Correlation Analysis Between the Gut Microbiota and the Metabolites, the Cytokines, the Endocrine Peptides, the Growth Performance

**FIGURE 6:**
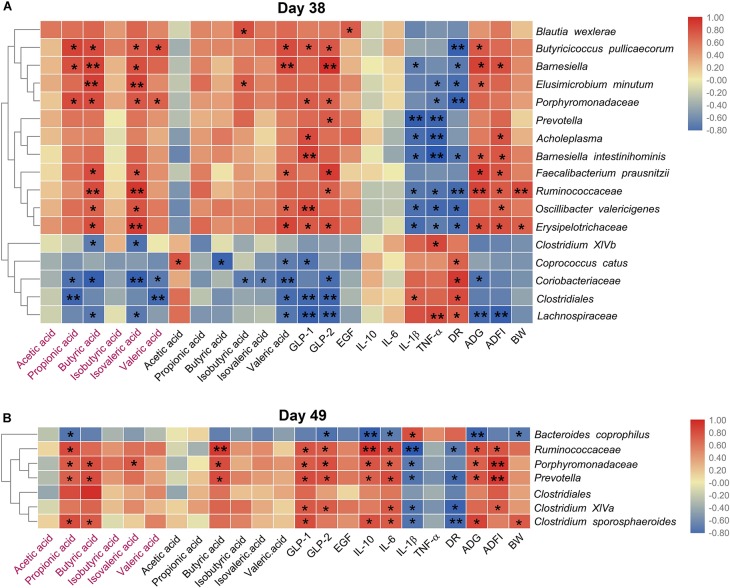
Heat map of the spearman’s rank correlation coefficient between the microbial species and the growth performance, the metabolites, the cytokines, endocrine peptides, the growth performance on day 38 **(A)** and day 49 **(B)**, respectively. In the figure, the blue color represents the negative correlation while red color represents a positive correlation. The words in red represents the SCFAs concentration of colonic digesta, and the words in normal black represents the SCFAs concentration in ileal digesta. Significance were presented as *P < 0.05, and **P < 0.01, *n* = 4 or 5.

We then explored the correlation between the species with different relative abundance and the piglets’ growth performance, the gut microbial metabolites, the serum cytokines. Figure 6A showed that on day 38, the relative abundances of 12 species (*Blautia wexlerae*, *Butyricicoccus pullicaecorum*, *Porphyromonadaceae*, *Elusimicrobium minutum*, *Barnesiella*, *Ruminococcaceae*, *Oscillibacter valericigenes*, *Erysipelotrichaceae*, *Acholeplasma*, *Prevotella*, *Faecalibacterium prausnitzii*, and *Barnesiella intestinihominis*) increased in Gly-Gln group, and the increase was positively associated with the growth performance (ADG, ADFI, and BW), and SCFAs (propionic acid, butyric acid, isovaleric acid, and valeric acid), but negatively associated with DR, IL-1β, and TNF-α. On the other hand, the relative abundances of other five species including *Coprococcus catus*, *Clostridiales*, *Coriobacteriaceae*, *Clostridium XlVb*, and *Lachnospiraceae* reduced in Gly-Gln group, and the decrease was negatively associated with the growth performance (ADG, ADFI, and BW), and SCFAs (propionic acid, butyric acid, isovaleric acid, and valeric acid), but positively associated with DR, IL-1β, and TNF-α. Later on day 49 (Figure 6B), the relative abundances of six species (*Ruminococcaceae*, *Porphyromonadaceae*, *Prevotella*, *Clostridium sporosphaeroides*, *Clostridium XlVa*, and *Clostridiales*) increased in Gly-Gln group, and the increase was positively associated with the growth performance (ADG, ADFI, and BW), and SCFAs (propionic acid, butyric acid, isovaleric acid, and valeric acid), but negatively associated with DR, IL-1β, and TNF-α. On the other hand, there was only one species *Bacteroides coprophilus* with decreased relative abundance in Gly-Gln group, and the decrease was negatively associated with the growth performance (ADG, ADFI, and BW), and SCFAs (propionic acid, butyric acid, isovaleric acid, and valeric acid), but positively associated with DR, IL-1β, and TNF-α. Most of these association were statistically significant (*P* < 0.05).

## Discussion

Firstly, we re-evaluated the beneficial effect of dietary Gly-Gln supplementation on the weaning transition of piglets. We confirmed that dietary 0.25% Gly-Gln supplementation notably elevated the villus height and increased villus height/crypt depth ratio, both of which indicate an improved intestinal function (Lallès et al., 2007). This beneficial effect was generally owed to Gly-Gln serving directly as a metabolic resource for the small intestine (Smith and Macfarlane, 1997; Lallès et al., 2007). At the same time, the inflammation response of weaning piglets was improved evidenced by decreased cytokines IL-1β, TNF-α, and IL-6, and increased cytokine IL-10 at the same time. The anti-inflammation effect of dietary Gln supplementation by maintaining mTOR and suppressing TLR4 and NOD signaling was also observed in weaning piglets challenged with lipopolysaccharide (Qin et al., 2018). Ultimately, the beneficial effect of dietary Gly-Gln supplementation was prominently displayed in reduced incidence of post-weaning diarrhea and enhanced growth performance of piglets including increased ADG, ADFI, and body weight.

In general, gut microbiota metabolize both exogenous and endogenous substrates into nutrients that can be used directly by the host (Oliphant and Allen-Vercoe, 2019). In addition, the effect of dietary supplementation with special protein and amino acid on gut microbiota is of growing interest (Fan et al., 2015; Liang et al., 2018; Oliphant and Allen-Vercoe, 2019). In this study, we found that dietary Gly-Gln supplementation can significantly shift piglets’ gut microbiota during weaning transition. 16S rDNA high-throughput sequencing analysis revealed that dietary Gly-Gln supplementation increased gut bacterial loading and alpha diversity in a time dependent manner. A considerable number of OTU selectively responded to Gly-Gln, which indicates that those OTU-assigned bacteria were enriched by dietary Gly-Gln supplementation. Similarly, dietary L-Tryptophan supplementation enhanced piglets’ growth performance and markedly altered their intestinal microbial composition, as is evidenced by enhanced alpha and beta diversity of gut microbiota (Liang et al., 2018). Moreover, Zhang et al. (2018) found that dietary L-arginine supplementation promoted the intestinal health and modulated the ileal microbiota of broiler chickens by enriching helpful bacteria and suppressing harmful species. Furthermore, alpha diversity analysis based on the OTUs revealed that piglets fed with dietary Gly-Gln supplementation harbored gut microbiota with significantly increased alpha diversity on day 38, but this increase outcomes was a little reduced on day 49. This indicated that dietary Gly-Gln supplementation at an earlier age of piglets may gain better beneficial effect on their gut microbiota. On the other hand, the early weaning stress will recover naturally in 8–14 days, which may relatively reduce the beneficial effect of dietary Gly-Gln supplementation (Sève, 2000; Gresse et al., 2017). Gresse et al. (2017) revealed that gut lumen niches where gut bacteria reside can be disturbed as a result of intestinal integrity damage in piglets suffering from weaning transition, which generally result in gut microbiota dysbiosis characterized by decreased bacteria diversity. The observed improvement in jejunal epithelial morphology of dietary Gly-Gln supplementation fed piglets may have resulted from the improvement of gut microbiota. An overall view of the gut microbiota shifts after dietary Gly-Gln supplementation provided by beta diversity analysis showed that the piglets’ gut microbiota was markedly altered on day 38 as well as on day 49. This gut microbiota-shift effect was also reported in obese adults after oral supplementation with L-glutamine (Zambom de Souza et al., 2015), and in post-weaned rabbits fed diets supplemented with 1.0% Gln (Chamorro et al., 2010). Furthermore, the gut microbiota-shift effect of amino acid was observed for other amino acids such as L-arginine and L-tryptophan (Liang et al., 2018; Zhang et al., 2018). Thereby, dietary Gly-Gln supplementation markedly shifted the gut microbiota of weaning piglets.

To figure out the bacteria responding to dietary Gly-Gln supplementation, we further assigned the OTUs to certain bacteria species. The analysis at the phylum level showed that dietary Gly-Gln supplementation increased the relative abundance of Fibrobacteres and Bacteroidetes, whereas decreased the relative abundance of Firmicutes on day 38. Consistent with this, dietary 1.0% Gln supplementation markedly increased the abundance of intestinal-friendly microbiota Bacteroidetes from gestational day 70–84 in sows (Zhang et al., 2017). Furthermore, the ratio of Firmicutes to Bacteroidetes was reduced in the gut microbiota of obese and overweight human when oral supplemented with L-glutamine (Zambom de Souza et al., 2015). The bacteria species of Fibrobacteres generally can be found in the gut of termites, ants, and even rumen of the ruminant animal where they digest cellulose to produce SCFAs in a strictly anaerobic environment (Varel et al., 1984; Metzler and Mosenthin, 2008). Notably, there are also many Fibrobacteres strains isolated from pig hindgut and feces (Varel et al., 1984). SCFAs produced by gut microbiota fermentation can benefit the host in numerous aspects. For instance, butyrate enhances the intestinal barrier and acetate mediates a microbiome–brain–β-cell axis to ameliorate metabolic syndrome (Peng et al., 2009; Perry et al., 2016). Given that Fibrobacteres with increased relative abundance may contribute to Gly-Gln’s beneficial effect on weaning piglets. Furthermore, the bacteria species of Bacteroidetes mainly produces acetate and propionic acid, whereas the bacteria species of Firmicutes has butyric acid as its primary metabolic terminal product (van den Berg et al., 2005). Dietary Gly-Gln supplementation may modify metabolic profiles of gut microbiota. In more detail, at species level dietary Gly-Gln supplementation increased the relative abundance of 12 species (*Barnesiella intestinihominis*, *Ruminococcaceae, Blautia wexlerae*, *Erysipelotrichaceae*, *Oscillibacter valericigenes*, *Elusimicrobium minutum*, *Porphyromonadaceae*, *Prevotella*, *pullicaecorum*, *Barnesiella*, *Acholeplasma*, and *Faecalibacterium prausnitzii*) on day 38 and six species (*Prevotella*, *Ruminococcaceae*, *Clostridium XlVa*, *Clostridiales*, and *Porphyromonadaceae*) on day 49. On the contrary, dietary Gly-Gln supplementation reduced the relative abundance of 5 species (*Clostridiales*, *Coprococcus catus*, *Coriobacteriaceae*, *Clostridium XlVb*, and *Lachnospiraceae*) on day 38 and one species *Bacteroides coprophilus* on day 49. Surprisingly, these species are all anaerobic except *Erysipelotrichaceae* and *Erysipelotrichaceae* with unknown metabolic type, indicating that dietary Gly-Gln supplementation mainly modulated the anaerobic bacteria in the hindgut. The metabolites of anaerobic fermentation by anaerobes mediate the interaction between gut microbiota and the host. For instance, butyric acid serves as the main energy resource of the colonic epithelial cell (Serino, 2019). However, early weaning piglets generally suffer the dysbiosis of gut microbiota induced by changes in diet and environment, which has increasingly been proved to be one of the main causes of post-weaning diarrhea (Gresse et al., 2017). The damaged intestinal epithelium will destroy the anaerobic environment and in turn facilitate the proliferation of facultative anaerobes such as *E. coli* (Gresse et al., 2017). Gut microbiota dysbiosis in mammals has been defined as a gut microbial imbalance identified by a marked decrease in the representation of obligate anaerobic bacteria, such as members of the classes *Clostridia* (Winter et al., 2013). In this study, we found that dietary Gly-Gln supplementation increased anaerobic bacteria, indicating that the dysbiosis was improved.

In the hindgut, mainly colon, the three macronutrients carbohydrates, proteins, and fat that have escaped primary digestion undergo anaerobic fermentation to produce metabolites such as SCFAs (Oliphant and Allen-Vercoe, 2019). Our study revealed that dietary Gly-Gln supplementation enriched the confirmed SCFAs-producing bacteria *Butyricicoccus pullicaecorum* (Eeckhaut et al., 2008), *Faecalibacterium prausnitzii* (Sokol et al., 2008; Miquel et al., 2013), and *Oscillibacter valericigenes* (Iino et al., 2007). We then determined the SCFAs concentration in colon and ileum and found that dietary Gly-Gln supplementation increased the concentration of propionic acid, isobutyric acid, and valeric acid on day 38, and increased the concentration of butyric acid on day 49 in digesta from colon and ileum of piglets. In a similar way, a previous study reported that dietary L-tryptophan supplementation enriched SCFAs-producing bacteria as well as their metabolites SCFAs in piglets (Liang et al., 2018). The SCFAs such as propionic acid and butyric acid are main final products from anaerobic fermentation and play a vital role not only in providing energy to the colonic epithelial cells, but also in regulating intestinal physiology, intestinal development, and nutrient absorption (Kasubuchi et al., 2015). Interestingly, valeric acid and isobutyric acid are either required by or stimulate the growth of many rumen organisms and are required for cellulose digestion (Muller, 1987; Yang, 2002). Valeric acid and isobutyric acid as branched-chain fatty acids (BCFA) are normally produced in the rumen as part of protein degradation (Russell and Hespell, 1981). Therefore, the increased valeric acid and isobutyric acid may favor the growth and proliferation of other cellulose-degrading bacteria in piglets’ hindgut. These postulations were supported by the significantly positive association between the SCFAs and the anaerobes with increased relative abundance on both day 38 and day 49. In addition, SCFAs showed regulation effects in the inflammatory responses (Vinolo et al., 2011). Collectively, dietary Gly-Gln supplementation improved the gut microbiota in piglets and increased the concentration of microbial metabolites SCFAs.

Recently, SCFAs have been shown to be ligands for two orphan G protein-coupled receptors (GPCRs) of G protein coupled receptor 41 (GPR41) and G protein coupled receptor 43 (GPR43), which regulate the level of various endocrine peptides (Miyamoto et al., 2016; Zhao et al., 2018). These endocrine peptides facilitate the proliferation, differentiation, and apoptosis of intestinal epithelial cells via regulating the secretion of the digestive glands and being involved in the processes of glycolysis and protein synthesis (Dubé and Brubaker, 2007; Rowland et al., 2011; Everard and Cani, 2014). A previous study demonstrated that elevated SCFAs concentration can stimulate the secretion of intestinal endocrine peptides including GLP-1, GLP-2, and EGF (Nohr et al., 2013). In this study, we determined these three endocrine peptides and found that dietary Gly-Gln supplementation increased the concentrations of GLP-1 and GLP-2 on both day 38 and day 49. A similar increase was also observed for EGF on day 48 though the trend of increase seemed to have slowed down on day 49. SCFAs can induce hormone GLP-1 release through activating GPR41 and GPR43 that act as co-sensors for SCFAs in enteroendocrine cells (Nohr et al., 2013). Of SCFAs, butyrate appears to be responsible for increasing plasma GLP-2 concentration through stimulating release of glucagon-like peptide-2 (GLP-2) from enteroendocrine L cells (Tappenden et al., 2003). Therefore, SCFAs produced by increased anaerobic microbial fermentation may have been responsible for activating GPCRs to stimulate the secretion of intestinal endocrine peptides in this study. The correlation analysis confirmed the relationship between bacteria and endocrine peptides in the ileum. We then explored the relationship of species that differ in relative abundance with the metabolites, the cytokines, the endocrine peptides, the growth performance using spearman’s rank correlation coefficient and significance test. We found obvious relevance between the indices and the species responding to dietary Gly-Gln supplementation. The results therefore showed that modulation of gut microbiota is an important factor in Gly-Gln’s favorable effect on piglets’ weaning-transition although the underlying mechanism remain unelucidated.

## Conclusion

In this study, we found that dietary Gly-Gln supplementation had beneficial effects on gut microbiota composition of piglets from day 28 to day 49 evidenced by increased bacterial loading, elevated alpha diversity, and increased proportions of anaerobes and fiber-degrading bacteria. Therefore, the increased SCFAs concentrations, as well as the endocrine peptides in the colon and ileum may contribute to the pro-weaning transition effect of dietary Gly-Gln supplementation in piglets. These findings will facilitate the improvement of gut microbiota targeted approaches to improve weaning transition of piglets by dietary functional amino acid.

## Data Availability Statement

The datasets generated for this study can be found in the NCBI accession PRJNA596825.

## Ethics Statement

The animal study was reviewed and approved by the Institutional Animal Care and Use Committee (IACUC) of Huazhong Agricultural University (Wuhan, China).

## Author Contributions

LM and XY designed the research. XX, YN, YT, XW, CX, TY, and SZ conducted the research. YY, BX, and BY analyzed the data. BX, YY, and LM wrote the manuscript and had responsibility for the final content. All authors read and approved the final manuscript.

## Conflict of Interest

The authors declare that the research was conducted in the absence of any commercial or financial relationships that could be construed as a potential conflict of interest.

## Supplementary Material

FIGURE S1 Dietary Gly-Gln supplementation improved jejunum morphology. Morphology of jejunum epithelium (H&E) at a magnification of 40 and 200 on day 38 and day 49.

TABLE S1 Ingredients and nutrients composition of the basal diet offered to piglets.

TABLE S2 Villus height, crypt depth and villus height/crypt depth ratio in the jejunum of piglets.

TABLE S3 Growth performance parameters of piglets.

## References

[B1] Barba-Vidal E. Castillejos L. Roll V. F. B. Cifuentes-Orjuela G. Moreno Muñoz J. A. Martín-Orúe S. M. (2017). The probiotic combination of *Bifidobacterium longum* subsp. *infantis* CECT 7210 and *Bifidobacterium animalis* subsp. *lactis* BPL6 reduces pathogen loads and improves gut health of weaned piglets orally challenged with *Salmonella* Typhimurium. *Front. Microbiol.* 8:1570 10.3389/fmicb.2017.01570 PMC5559543 28861074

[B2] Belkaid Y. Hand T. W. (2014). Role of the microbiota in immunity and inflammation. *Cell.* 157 121–141. 10.1016/j.cell.2014.03.01 24679531 PMC4056765

[B3] Bolyen E. Rideout J. R. Dillon M. R. Bokulich N. A. Abnet C. C. Al-Ghalith G. A. (2019). Reproducible, interactive, scalable and extensible microbiome data science using QIIME 2. *Nat. Biotechnol.* 37 852–857. 31341288 10.1038/s41587-019-0209-9 PMC7015180

[B4] Chamorro S. De Blas C. Grant G. Badiola I. Menoyo D. Carabaño R. (2010). Effect of dietary supplementation with glutamine and a combination of glutamine-arginine on intestinal health in twenty-five-day-old weaned rabbits. *J. Anim. Sci.* 88 170–180. 10.2527/jas.2008-1698 19783707

[B5] Chassard C. Dapoigny M. Scott K. P. Crouzet L. Del’homme C. Marquet P. (2012). Functional dysbiosis within the gut microbiota of patients with constipated−irritable bowel syndrome. *Aliment. Pharmacol. Ther.* 35 828–838. 10.1111/j.1365-2036.2012.05007.x 22315951

[B6] Chen H. Mao X. He J. Yu B. Huang Z. Yu J. (2013). Dietary fibre affects intestinal mucosal barrier function and regulates intestinal bacteria in weaning piglets. *Br. J. Nutr.* 110 1837–1848. 10.1017/s0007114513001293 23656640

[B7] Costea P. I. Zeller G. Sunagawa S. Pelletier E. Alberti A. Levenez F. (2017). Towards standards for human fecal sample processing in metagenomic studies. *Nat. Biotechnol..* 35 1069–1076. 10.1038/nbt.3960 28967887

[B8] Dai Z.-L. Li X.-L. Xi P.-B. Zhang J. Wu G. Zhu W.-Y. (2013). l-Glutamine regulates amino acid utilization by intestinal bacteria. *Amino. Acids* 45 501–512. 10.1007/s00726-012-1264-1264 22451274

[B9] David L. A. Maurice C. F. Carmody R. N. Gootenberg D. B. Button J. E. Wolfe B. E. (2013). Diet rapidly and reproducibly alters the human gut microbiome. *Nature* 505 559–563. 10.1038/nature12820 24336217 PMC3957428

[B10] DeSantis T. Z. Hugenholtz P. Larsen N. Rojas M. Brodie E. L. Keller K. (2006). Greengenes, a chimera-checked 16S rRNA gene database and workbench compatible with ARB. *Appl. Environ. Microbiol.* 72 5069–5072. 10.1128/aem.03006-3005 16820507 PMC1489311

[B11] Dubé P. E. Brubaker P. L. (2007). Frontiers in glucagon-like peptide-2: multiple actions, multiple mediators. *Am. J. Physiol. -Endocrinol. Metab.* 293 E460–E465. 10.1152/ajpendo.00149.2007 17652153

[B12] Edgar R. C. (2013). UPARSE: highly accurate OTU sequences from microbial amplicon reads. *Nat. Methods* 10 996–998. 10.1038/nmeth.260 23955772

[B13] Eeckhaut V. Van Immerseel F. Teirlynck E. Pasmans F. Fievez V. Snauwaert C. (2008). *Butyricicoccus pullicaecorum* gen. nov., sp. nov., an anaerobic, butyrate-producing bacterium isolated from the caecal content of a broiler chicken. *Int. J. Syst. Evol. Microbiol.* 58 2799–2802. 10.1099/ijs.0.65730-65730 19060061

[B14] Everard A. Cani P. D. (2014). Gut microbiota and GLP-1. *Rev. Endocr. Metab. Disord.* 15 189–196. 10.1007/s11154-014-9288-9286 24789701

[B15] Fadrosh D. W. Ma B. Gajer P. Sengamalay N. Ott S. Brotman R. M. (2014). An improved dual-indexing approach for multiplexed 16S rRNA gene sequencing on the Illumina MiSeq platform. *Microbiome* 2 1–7. 10.1186/2049-2618-2-6 24558975 PMC3940169

[B16] Fan P. X. Li L. S. Rezaei A. Eslamfam S. Che D. S. Ma X. (2015). Metabolites of Dietary Protein and Peptides by Intestinal Microbes and their Impacts on Gut. *Curr. Prot. Peptide Sci.* 16 646–654. 10.2174/1389203716666150630133657 26122784

[B17] Franklin M. A. Mathew A. G. Vickers J. R. Clift R. A. (2002). Characterization of microbial populations and volatile fatty acid concentrations in the jejunum, ileum, and cecum of pigs weaned at 17 vs 24 days of age. *J. Anim. Sci.* 80 2904–2910. 10.2527/2002.80112904x 12462258

[B18] Gillis C. C. Hughes E. R. Spiga L. Winter M. G. Zhu W. Furtado de Carvalho T. (2018). Dysbiosis-associated change in host metabolism generates lactate to support *Salmonella* growth. *Cell Host Microbe* 23 54–64. 10.1016/j.chom.2017.11.006 29276172 PMC5764812

[B19] Gong J. Yu H. Liu T. Li M. Si W. de Lange C. F. (2008). Characterization of ileal bacterial microbiota in newly-weaned pigs in response to feeding lincomycin, organic acids or herbal extract. *Livestock Sci.* 116 318–322. 10.1016/j.livsci.2008.01.001

[B20] Gresse R. Chaucheyras-Durand F. Fleury M. A. Van de Wiele T. Forano E. Blanquet-Diot S. (2017). Gut microbiota dysbiosis in postweaning piglets: understanding the keys to Health. *Trends Microbiol.*. 25 851–873. 10.1016/j.tim.2017.05.004 28602521

[B21] Horler D. Westlake D. McConnell W. (1966). Conversion of glutamic acid to volatile acids by Micrococcus aerogenes. *Can. J. Microbiol.* 12 47–53. 10.1139/m66-008 5923136

[B22] Iino T. Mori K. Tanaka K. Suzuki K.-I. Harayama S. (2007). Oscillibacter valericigenes gen. nov., sp. nov., a valerate-producing anaerobic bacterium isolated from the alimentary canal of a Japanese corbicula clam. *Int. J. Syst. Evol. Microbiol.* 57(pt 8), 1840–1845. 10.1099/ijs.0.64717-64710 17684268

[B23] Jiang J.-W. Ren Z.-G. Chen L.-Y. Jiang L. Xie H.-Y. Zhou L. (2011). Enteral supplementation with glycyl-glutamine improves intestinal barrier function after liver transplantation in rats. *Hepatobiliary Pancreat. Dis. Int.* 10 380–385. 10.1016/S1499-3872(11)60064-60067 21813386

[B24] Jiang Z. Sun L. Lin Y. Ma X. Zheng C. Zhou G. (2009). Effects of dietary glycyl-glutamine on growth performance, small intestinal integrity, and immune responses of weaning piglets challenged with lipopolysaccharide. *J. Anim. Sci.* 87 4050–4056. 10.2527/jas.2008-1120 19717785

[B25] Jiang J. Li J. Li Y. Wang X. Wang Z. Liu F. (2000). The dipeptide glycyl-glutamine enhances absorptive function of autotransplanted small intestine in pig. *Parenter. Enteral Nutr.* Available at: http://en.cnki.com.cn/Article_en/CJFDTotal-CWCN200002006.htm

[B26] Jiao L. F. Song Z. H. Ke Y. L. Xiao K. Hu C. H. Shi B. (2014). Cello-oligosaccharide influences intestinal microflora, mucosal architecture and nutrient transport in weaned pigs. *Anim. Feed Sci. Technol.* 195 85–91. 10.1016/j.anifeedsci.2014.05.014

[B27] Kasubuchi M. Hasegawa S. Hiramatsu T. Ichimura A. Kimura I. (2015). Dietary gut microbial metabolites, short-chain fatty acids, and host metabolic regulation. *Nutrients* 7 2839–2849. 10.3390/nu7042839 25875123 PMC4425176

[B28] Kim Y.-G. Udayanga K. G. Totsuka N. Weinberg J. B. Núñez G. Shibuya A. (2014). Gut dysbiosis promotes M2 macrophage polarization and allergic airway inflammation via fungi-induced PGE_2_. *Cell Host Microbe* 15 95–102. 10.1016/j.chom.2013.12.010 24439901 PMC3957200

[B29] Lallès J.-P. Bosi P. Smidt H. Stokes C. R. (2007). Nutritional management of gut health in pigs around weaning. *Proc. Nutr. Soc.* 66 260–268. 10.1017/S0029665107005484 17466106

[B30] Li D. F. Thaler R. C. Nelssen J. L. Harmon D. L. Allee G. L. Weeden T. L. (1990). Effect of fat sources and combinations on starter pig performance. Nutrient digestibility and intestinal morphology. *J. Anim. Sci.* 68 3694–3704. 2262422 10.2527/1990.68113694x

[B31] Li Y. Li J. Jiang J. Li N. Wang X. Wang Z. (2003). Glycyl-glutamine-supplemented long-term total parenteral nutrition selectively improves structure and function in heterotopic small-bowel autotransplantation in the pig. *Transpl. Int.* 16 866–871. 10.1007/s00147-003-0645-648 12915960

[B32] Liang H. Dai Z. Liu N. Ji Y. Chen J. Zhang Y. (2018). Dietary L-tryptophan modulates the structural and functional composition of the intestinal microbiome in weaned piglets. *Front. Microbiol.* 9:1736. 10.3389/fmicb.2018.01736 30131777 PMC6090026

[B33] Magoč T. Salzberg S. L. (2011). FLASH: fast length adjustment of short reads to improve genome assemblies. *Bioinformatics* 27 2957–2963. 10.1093/bioinformatics/btr507 21903629 PMC3198573

[B34] Martin R. Nauta A. Ben Amor K. Knippels L. Knol J. Garssen J. (2010). Early life: gut microbiota and immune development in infancy. *Beneficial. Microbes* 1 367–382. 10.3920/BM2010.0027 21831776

[B35] Metzler B. U. Mosenthin R. (2008). A review of interactions between dietary fiber and the gastrointestinal microbiota and their consequences on intestinal phosphorus metabolism in growing pigs. *AsianAust. J. Anim. Sci.* 21 603–615. 10.5713/ajas.2008.r.03

[B36] Micah H. Claire F.-L. Rob K. J. N. (2007). The human microbiome project: exploring the microbial part of ourselves in a changing world. *Nature* 449 804–810. 17943116 10.1038/nature06244 PMC3709439

[B37] Miquel S. Martín R. Rossi O. Bermúdez-Humarán L. G. Chatel J. M. Sokol H. (2013). Faecalibacterium prausnitzii and human intestinal health. *Curr. Opin. Microbiol.* 16 255–261. 10.1016/j.mib.2013.06.003 23831042

[B38] Miyamoto J. Hasegawa S. Kasubuchi M. Ichimura A. Nakajima A. Kimura I. (2016). Nutritional signaling via free fatty acid receptors. *Int. J. Mol. Sci.* 17:450. 10.3390/ijms17040450 27023530 PMC4848906

[B39] Muller L. D. (1987). Branched chain fatty acids (Isoacids) and valeric acid for ruminants12. *Prof. Anim. Sci.* 3 9–12. 10.15232/S1080-7446(15)32370-32376

[B40] National Research Council [NRC] (2012). *Nutrient Requirements of Swine*, 11th Edn. Washington, DC: National Academies Press 10.17226/13298

[B41] Nohr M. K. Pedersen M. H. Gille A. Egerod K. L. Engelstoft M. S. Husted A. S. (2013). GPR41/FFAR3 and GPR43/FFAR2 as cosensors for short-chain fatty acids in enteroendocrine cells vs FFAR3 in enteric neurons and FFAR2 in enteric leukocytes. *Endocrinology* 154 3552–3564. 10.1210/en.2013-1142 23885020

[B42] Oliphant K. Allen-Vercoe E. (2019). Macronutrient metabolism by the human gut microbiome: major fermentation by-products and their impact on host health. *Microbiome* 7 91. 10.1186/s40168-019-0704-708 31196177 PMC6567490

[B43] Parks D. H. Tyson G. W. Hugenholtz P. Beiko R. G. (2014). STAMP: statistical analysis of taxonomic and functional profiles. *Bioinformatics* 30 3123–3124. 10.1093/bioinformatics/btu494 25061070 PMC4609014

[B44] Peng L. Y. Li Z. R. Green R. S. Holzman I. R. Lin J. (2009). Butyrate enhances the intestinal barrier by facilitating tight junction assembly via activation of AMP-activated protein kinase in Caco-2 cell monolayers. *J. Nutr.* 139 1619–1625. 10.3945/jn.109.104638 19625695 PMC2728689

[B45] Perry R. J. Peng L. Barry N. A. Cline G. W. Zhang D. Cardone R. L. (2016). Acetate mediates a microbiome–brain–β-cell axis to promote metabolic syndrome. *Nature* 534 213–217. 10.1038/nature18309 27279214 PMC4922538

[B46] Qin Q. Xu X. Wang X. Wu H. Zhu H. Hou Y. (2018). Glutamate alleviates intestinal injury, maintains mTOR and suppresses TLR4 and NOD signaling pathways in weanling pigs challenged with lipopolysaccharide. *Sci. Rep.* 8:15124. 10.1038/s41598-018-33345-33347 30310102 PMC6181909

[B47] Ren W. Chen S. Yin J. Duan J. Li T. Liu G. (2014). Dietary arginine supplementation of mice alters the microbial population and activates intestinal innate immunity. *J. Nutr.* 144 988–995. 10.3945/jn.114.192120 24670969

[B48] Rowland K. J. Trivedi S. Lee D. Wan K. Kulkarni R. N. Holzenberger M. (2011). Loss of glucagon-like peptide-2-induced proliferation following intestinal epithelial insulin-like growth factor-1-receptor deletion. *Gastroenterology* 141 2166.e7–2175.e7. 10.1053/j.gastro.2011.09.014 21925122

[B49] Russell J. B. Hespell R. B. (1981). Microbial rumen fermentation. *J. Dairy Sci* 64 1153–1169. 10.3168/jds.s0022-0302(81)82694-x 7024344

[B50] Schloss P. Westcott S. Ryabin T. Hall J. Hartmann M. Hollister E. (2009). Introducing mothur: open-source, platform-independent, community-supported software for describing and comparing microbial communities. *Appl. Environ. Microbiol.* 75 7537–7541. 10.1128/AEM.01541-09 19801464 PMC2786419

[B51] Serino M. (2019). SCFAs — the thin microbial metabolic line between good and bad. *Nat. Rev. Endocrinol..* 15 318–319. 10.1038/s41574-019-0205-207 30976118

[B52] Sève B. (2000). Effects of underfeeding during the weaning period on growth, metabolism, and hormonal adjustments in the piglet. *Domest. Anim. Endocrinol.* 19 63–74. 10.1016/S0739-7240(00)00067-69 11025186

[B53] Smith E. A. Macfarlane G. J. A. (1997). Dissimilatory amino acid metabolism in human colonic bacteria. *Anaerobe.* 3 327–337. 10.1006/anae.1997.0121 16887608

[B54] Soderborg T. K. Clark S. E. Mulligan C. E. Janssen R. C. Babcock L. Ir D. (2018). The gut microbiota in infants of obese mothers increases inflammation and susceptibility to NAFLD. *Nat. Commun.* 9:4462. 10.1038/s41467-018-06929-6920 30367045 PMC6203757

[B55] Sokol H. Pigneur B. Watterlot L. Lakhdari O. Bermúdez-Humarán L. G. Gratadoux J.-J. (2008). Faecalibacterium prausnitzii is an anti-inflammatory commensal bacterium identified by gut microbiota analysis of Crohn disease patients. *Proc. Natl. Acad. Sci. U.S.A.* 105:16731. 10.1073/pnas.0804812105 18936492 PMC2575488

[B56] Tappenden K. A. Albin D. M. Bartholome A. L. Mangian H. F. (2003). Glucagon-like peptide-2 and short-chain fatty acids: a new twist to an old story. *J Nutr* 133 3717–3720. 10.1093/jn/133.11.3717 14608102

[B57] van den Berg A. van Elburg R. M. Westerbeek E. A. Twisk J. W. Fetter W. P. (2005). Glutamine-enriched enteral nutrition in very-low-birth-weight infants and effects on feeding tolerance and infectious morbidity: a randomized controlled trial. *Am. J. Clin. Nutr.* 81 1397–1404. 10.1093/ajcn/81.6.1397 15941893

[B58] Varel V. H. Fryda S. J. Robinson I. M. (1984). Cellulolytic bacteria from pig large intestine. *Appl. Environ. Microbiol.* 47 219–221. 6696420 10.1128/aem.47.1.219-221.1984 PMC239643

[B59] Vinolo M. A. Rodrigues H. G. Nachbar R. T. Curi R. J. N. (2011). Regulation of inflammation by short chain fatty acids. *Nutrients* 3 858–876. 10.3390/nu3100858 22254083 PMC3257741

[B60] Wada S. Sato K. Ohta R. Wada E. Bou Y. Fujiwara M. (2013). Ingestion of low dose pyroglutamyl leucine improves dextran sulfate sodium-induced colitis and intestinal microbiota in mice. *J. Agric Food Chem.* 61 8807–8813. 10.1021/jf402515a 23964746

[B61] Wang J. J. Chen L. X. Li P. Li X. L. Zhou H. J. Wang F. L. (2008). Gene expression is altered in piglet small intestine by weaning and dietary glutamine supplementation. *J. Nutr.* 138 1025–1032. 10.1093/jn/138.6.1025 18492829

[B62] Winter S. E. Winter M. G. Xavier M. N. Thiennimitr P. Poon V. Keestra A. M. (2013). Host-derived nitrate boosts growth of *E. coli* in the inflamed gut. *Science* 339 708–711. 10.1126/science.1232467 23393266 PMC4004111

[B63] Xu C. Yang S. Zhu L. Cai X. Sheng Y. Zhu S. (2014). Regulation of N-acetyl cysteine on gut redox status and major microbiota in weaned piglets. *J. Anim. Sci.* 92 1504–1511. 10.2527/jas.2013-6755 24496840

[B64] Yang C. M. J. (2002). Response of forage fiber degradation by ruminal microorganisms to branched-chain volatile fatty acids, amino acids, and dipeptides. *J. Dairy Sci.* 85 1183–1190. 10.3168/jds.s0022-0302(02)74181-7 12086054

[B65] Yang K. M. Jiang Z. Y. Zheng C. T. Wang L. Yang X. F. (2014). Effect of Lactobacillus plantarum on diarrhea and intestinal barrier function of young piglets challenged with enterotoxigenic *Escherichia coli* K88. *J. Anim. Sci.* 92 1496–1503. 10.2527/jas.2013-6619 24492550

[B66] Yang Z. Huang S. Zou D. Dong D. He X. Liu N. (2016). Metabolic shifts and structural changes in the gut microbiota upon branched-chain amino acid supplementation in middle-aged mice. *Amino Acids* 48 2731–2745. 10.1007/s00726-016-2308-y 27539648

[B67] Zambom de Souza A. Z. Zambom A. Z. Abboud K. Y. Reis S. K. Tannihão F. Guadagnini D. (2015). Oral supplementation with l-glutamine alters gut microbiota of obese and overweight adults: a pilot study. *Nutrition* 31 884–889. 10.1016/j.nut.2015.01.004 25933498

[B68] Zeng Z. Xu X. Zhang Q. Li P. Zhao P. Li Q. (2015). Effects of essential oil supplementation of a low−energy diet on performance, intestinal morphology and microflora, immune properties and antioxidant activities in weaned pigs. *Anim. Sci. J.* 86 279–285. 10.1111/asj.12277 25302651

[B69] Zhang B. Lv Z. Li Z. Wang W. Li G. Guo Y. (2018). Dietary l-arginine supplementation alleviates the intestinal injury and modulates the gut microbiota in broiler chickens challenged by clostridium perfringens. *Front. Microbiol.* 9:1716. 10.3389/fmicb.2018.01716 30108569 PMC6080643

[B70] Zhang Y. Lu T. Han L. Zhao L. Niu Y. Chen H. (2017). L-Glutamine supplementation alleviates constipation during late gestation of mini sows by modifying the microbiota composition in feces. *Biomed. Res. Int..* 2017:4862861. 10.1155/2017/4862861 28386552 PMC5366184

[B71] Zhao Y. Chen F. Wu W. Sun M. Bilotta A. J. Yao S. (2018). GPR43 mediates microbiota metabolite SCFA regulation of antimicrobial peptide expression in intestinal epithelial cells via activation of mTOR and STAT3. *Mucosal Immunol.* 11 752–762. 10.1038/mi.2017.118 29411774 PMC5976519

[B72] Zhou P. Zhao Y. Zhang P. Li Y. Gui T. Wang J. (2017). Microbial mechanistic insight into the role of inulin in improving maternal health in a pregnant sow model. *Front. Microbiol.* 8:2242. 10.3389/fmicb.2017.02242 29204137 PMC5698696

