# Supplementary figures and images for: Modulation of Gut Microbial Community and Metabolism by Dietary Glycyl-Glutamine Supplementation May Favor Weaning Transition in Piglets
Source: Front Microbiol. 2020 Jan 28;10:3125. doi: 10.3389/fmicb.2019.03125 (PMC7025575; doi:10.3389/fmicb.2019.03125)

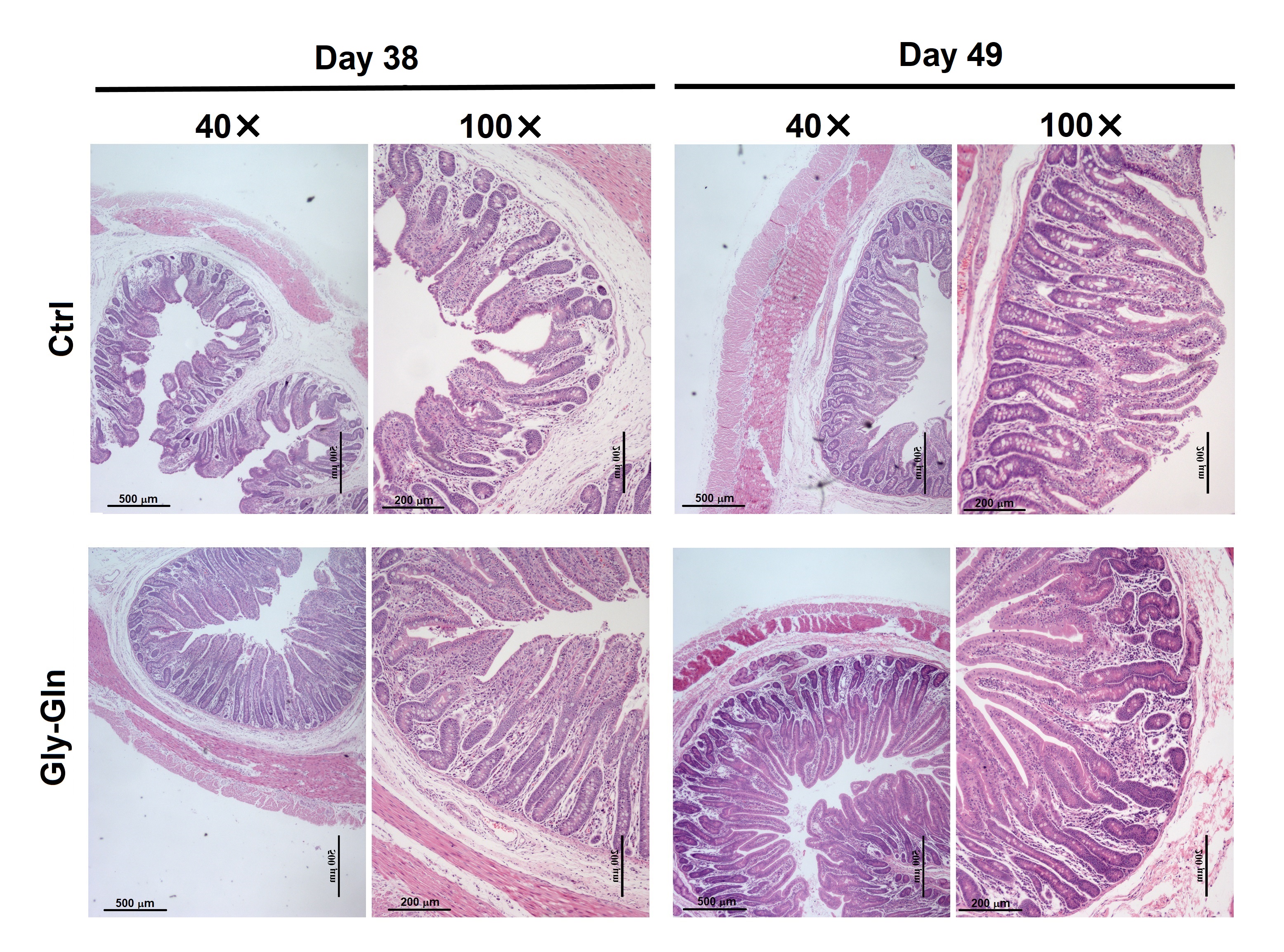

Supplement: FIGURE S1 — Dietary Gly-Gln supplementation improved jejunum morphology. Morphology of jejunum epithelium (H&E) at a magnification of 40 and 200 on day 38 and day 49. [file Image_1.jpeg]
