# Supplementary material for: Modulation of Gut Microbial Community and Metabolism by Dietary Glycyl-Glutamine Supplementation May Favor Weaning Transition in Piglets
Source: Front Microbiol. 2020 Jan 28;10:3125. doi: 10.3389/fmicb.2019.03125 (PMC7025575; doi:10.3389/fmicb.2019.03125)
Supplement: TABLE S1 — Ingredients and nutrients composition of the basal diet offered to piglets. [file Data_Sheet_1.PDF]

Supplementary Tables

TABLE S1. Ingredients and nutrients composition of the basal diet offered to piglets

Ingredients (%)	Postnatal d28-49
Corn	51.49
Soybean meal	11
Whey powder	5.0
Puffed rice noodles	5.0
Soy protein concentrate	4.0
Expanded soybeans	5.0
Fermented soybean meal	4.0
Soybean oil	2.5
Fish meal	3.0
ZnO	0.35
Stone powder	0.8
Sugar	1.5
Glucose	2.5
Acidifiers	0.35
Yeast nucleic acid	0.5
Tryptophane	0.04
$CaHPO_4$	1.1
Salt	0.25
DL-Met	0.08
L-Lys	0.42
L-Thr	0.12
Vitamin premix[1]	0.50
Mineral premix[2]	0.50
Total	100.00
Nutrient levels	
Crude protein (%)	18.36
Digestible energy (Mcal/kg)	13.03
Total Lys (%)	1.32
Total Met (%)	0.38
Total Met + Cys (%)	0.82
Calcium (%)	0.88
Total phosphorus (%)	0.68

[1]Vitamin premix provided per kg of diet: retinyl acetate, 10000 IU; cholecalciferol 2600 IU; dl-α-tocopheryl acetate, 55 IU; menadione, 6.0 mg; thiamin, 3.0 mg; riboflavin, 5.0 mg; pantothenic acid, 12.0 mg; pyridoxine, 10.0 mg; niacin, 30.0 mg; *d*-biotin, 0.2 mg; folic acid, 1.5 mg; cyanocobalamin, 0.05 mg; choline chloride 1600 mg.

[2]Mineral premix provided per kg of diet: $FeSO_4•7H_2O$, 500 mg; $CuSO_4•5H_2O$, 80.7 mg; $MnSO_4•5H_2O$, 120mg; $ZnSO_4•7H_2O$, 500 mg; Na_2SeO_3, 0.80mg; KI, 0.5 mg.

TABLE S2. Villus height, crypt depth and villus height/crypt depth ratio in the jejunum of piglets.

.

Item	Ctrl	Gly-Gln	*P*-value
**Villus height (mm)**			
Day 38	96.84±8.00	141.36±32.80	0.029
Day 49	115.83±19.04	165.88±15.62	0.0036
**Crypt depth (mm)**			
Day 38	145.18±36.00	64.87±7.55	0.0024
Day 49	122.24±23.12	98.79±19.81	0.1621
**Villus height / Crypt depth**			
Day 38	1.06±0.13	1.73±0.35	0.0069
Day 49	0.97±0.07	1.83±0.25	0.0002

TABLE S3. Growth performance parameters of piglets.

Item	Ctrl	Gly-Gln	*P*-value
**BW (kg)**			
Day 28	7.04±0.63	7.10±0.37	0.850
Day 38	9.41±1.10	10.81±1.10	0.072
Day 49	11.83±1.34	14.29±0.95	0.018
**ADFI (g/day)**			
Day 28 - Day 38	313.08±5.38	391.35±3.59	0.022
Day 38 - Day 49	389.03±5.13	461.02±3.93	0.032
**ADG (g/day)**			
Day 28 - Day 38	219.33±7.33	326.00±3.87	0.016
Day 38 - Day 49	120.67±3.09	173.61±3.57	0.031
**DR**			
Day 28 - Day 49	0.055±0.029	0.023±0.015	0.053

Ctrl, a control group; Gly-Gln, a 0.25 % Gly-Gln supplemented group. BW, body weight; ADFI, average daily feed intake; ADG, average daily gain; DR, diarrhea ratio.
